# Review of "Toxoplasmosis of Animals and Humans (Second Edition)" by J.P. Dubey

**DOI:** 10.1186/1756-3305-3-112

**Published:** 2010-11-23

**Authors:** Joanne P Webster

**Affiliations:** 1 Department of Infectious Disease Epidemiology, School of Public Health, St Mary's Hospital Campus, Imperial College Faculty of Medicine, London, W2 1PG, UK

## Review

The intracellular apicomplexan protozoan *Toxoplasma gondii *is found worldwide, is capable of infecting almost any cell type within an exceptionally broad host range - across humans, livestock, companion animals and wildlife, making it one of the most 'successful' protozoan parasites on earth. It has been just over 100 years since the discovery of *Toxoplasma gondii *parasite in 1908, in the little hamster-like rodent *Ctenodactylus gundi*, by Nicolle and Manceaux.

This book, 'Toxoplasmosis of Animals and Humans', marks almost two decades since the publication of its first edition, originally written by both the current author, J.P. Dubey, together with his former professor, the late C.P. Beattie. During this period there undoubtedly has been a proliferation of knowledge concerning *T. gondii *and toxoplasmosis, to the point where it is perhaps no longer the 'poor cousin' to malaria research. Indeed, even the genome of at least one *T. gondii *reference line has been published recently. Such an updated edition is thus certainly timely and will provide a valuable addition to all those biology, veterinary, medical researchers and students working in this field.

The book opens with a chapter on the general biology of *Toxoplasma gondii*. It considers the history of the parasite, including, for instance, its potential evolution from a coccidian parasite of cats with a faecal-oral cycle prior to subsequent adaptation to additional transmission routes through carnivorism and transplacental transmission. Recent developments in our understanding of the cell biology and molecular biology of the parasite are also briefly covered. Likewise, this chapter touches on the current knowledge concerning potential behavioural alterations associated with infection in both humans and animals and, in a little more detail, the host-parasite relationship in general. This introductory section has been updated to acknowledge that human adult-acquired infections previously thought to be 'asymptomatic may be more complex, albeit only rarely causing severe clinical manifestations. Indeed it also effectively covers what is now known, and what remains to be known, on the potential associations between parasite virulence and the relative roles of parasite strain, host variability including gender, the environment and the potential interactions between each. Prevention and control of infection in general is briefly introduced here. Finally, this opening chapter ends with invaluable details for laboratory techniques - from the cultivation, maintenance and infection routes recommended to the identification of cysts and descriptions of the range and relative advantages of serological tests currently available. The information provided in this chapter alone will thus provide essential reading for researchers setting up new studies, not to mention providing much of the health and safety documentation needed to support them!

Chapter 2 then focuses exclusively on toxoplasmosis in humans. As with each subsequent chapter, species by species, the worldwide seroprevalence reports are presented - here across components of the general population between countries as well as specifically relating to antibodies in pregnant women and/or women of child-bearing age. Clinical symptoms in relation to transmission route and/or host immunocompetence status are covered in detail, as are the various treatment options. This section is interspersed with several nice little snippets of information, such as when, for example, the author himself had acquired toxoplasmosis, and regarding the naming of the RH strain after the initials of the 6-old boy from Ohio from which it was originally isolated in 1937.

The subsequent chapters 3 to 19 cover toxoplasmosis in the range of studied animal species, from domestic cats and other felids, to that of 'miscellaneous animals' such as the Dik-Dik and Muskox. Each chapter reviews in detail the seroprevalence reports and transmission dynamics, covering, where available, natural and experimental infections. As each chapter within this book is written by the same author who is one of the leaders in this field over much of the last 45 years, the text follows in a consistent and highly readable manner. Almost 1400 key citations are provided, primarily from the 1988 to 2008 literature (notably 166 with J.P. Dubey as first author), which effectively guides the reader to further information where appropriate, and prime areas in need of future research are highlighted.

In summary, this new edition of 'Toxoplasmosis of Animals and Humans', very nicely illustrated throughout, often in colour, provides in a single volume a comprehensive and invaluable source of information regarding recent developments and current state-of-the-art in our understanding of *Toxoplasma gondii *basic biology, transmission, laboratory culture, and potential control. Focal aspects, such as the biology of the parasite and seroprevalence studies across host species are presented in detail, whilst the reader is led to suitable further reading for certain other topics. I have no hesitation in recommending this book highly.

## Competing interests

The author declares that they have no competing interests.

